# Supplementary material for: Aromatic side-chain conformational switch on the surface of the RNA Recognition Motif enables RNA discrimination
Source: Nat Commun. 2017 Sep 21;8:654. doi: 10.1038/s41467-017-00631-3 (PMC5608764; doi:10.1038/s41467-017-00631-3)
Supplement: Supplementary file 1 — Supplementary Information [file 41467_2017_631_MOESM1_ESM.pdf]

Description of Supplementary Files

File name: Supplementary Information
Description: Supplementary figures and supplementary tables.

File name: Peer review file

Supplementary Table 1: List of intermolecular hydrogen bonds observed in the bundle of 20 structures

RRM3/UUUAA

Donor-H	Acceptor	%
His429 N-HE2 [1)]	U1 O2′	50
His429 N-HE2 [1)]	U1 O2	35
Lys495 N-HZ3+ [2)]	U1 O2	65
His429 N-H	U2 OP1	35
Lys495 N-HZ3+ [2)]	U2 O4	35
U3 N-H3 [3)]	Leu498 O	40
Arg500 N-H	U3 O2	75
Arg500 N-HE	U3 O2′ [4)]	35
Arg500 N-HH*	U3 O2′ [4)]	25
U3 N-H3 [3)]	Arg500 N	45
A4 N-H61	Glu418 OE1/OE2	50
Lys453 N-HZ3+ [5)]	A4 N3	35
Gln416 N-HE22	A5 N1	30
Ile456 N-H	A5 O2′	90
Ile456 N-H	A5 O3′ [6)]	70
Lys464 N-HZ3+	A5 O3′ [6)]	70
Lys453 N-HZ3+ [5)]	A5 N3	40

RRM3/UGUGUG

Donor-H	Acceptor	%
Gln410 N-HE22	U1 O4	30
U1 N-H3	Ser441 O	25
Lys442 N-HZ3+	U1 O4'	75
Lys473 N-HZ3+	G2 N7	35
Arg478 N-HH*	G2 O3′	40
Gln475 N-HE22	U3 O4	35
Arg478 N-H	U3 O2	100
Arg478 N-HH*	U3 OP2	100
G4 N-H22	Ser393 O	85
G4 N-H21	Ser429 OG	75
G4 N-H21	Ser429 O	30
G4 N-H1	Ser448 OG	80
Lys477 N-HZ3+	G4 OP2	40
Lys477 N-HZ3+	G4 N7	60
Gln394 N-HE22	U5 OP2	80
Ile434 N-H	U5 O4	100
U5 N-H3	Ile434 O	100
Lys436 N-HZ3+	U5 O3′	40
Lys431 N-HZ3+	G6 O6	70
Lys431 N-HZ3+	G6 N7	25
Lys436 N-HZ3+	G6 OP1	25
Lys436 N-HZ3+	G6 O5′	35
G6 N-H22	Asn439 OD1	35

H-bond donors or acceptors that can form alternative H-bonds are marked with the same superscript number

Analyzed by MOLMOL: distance (H – Acceptor) ≤3.0 Å, angle (H – Donor – Acceptor) ≤ 45.0°, occurrence ≥ 25%.

N-HH*= N-HH12/N-HH22

Supplementary Table 2: ^{3}J couplings and $\chi 1$ rotamer populations: CUG-BP2 RRM3

	$^{3}J_{exp}$, NC^{γ}	$^{3}J_{exp}$, $C'C^{\gamma}$	$^{3}J_{calc}$, NC^{γ}	$^{3}J_{calc}$, $C'C^{\gamma}$	p_{-60}	p_{+60}	p_{180}
**RRM3 *wild type***							
F426	0.62±0.19	3.26±0.46	0.62	3.26	0.70	0.19	0.11
Y428	0.33±0.15	3.89±0.20	0.38	3.89	0.86	0.14	0.00
H429	0.60±0.06	5.09±0.05	0.50	4.97	1.00	0.00	0.00
F434	0.56±0.16	3.23±0.38	0.56	3.23	0.69	0.23	0.08
F443	0.22±0.11	4.57±0.12	0.38	4.43	1.00	0.00	0.00
F446	0.54±0.09	3.91±0.15	0.54	3.91	0.87	0.06	0.07
F455	n.d.	3.85±0.38		3.85	0.85	0.15	
F466	no peak	1.88±0.45		1.88	0.34	0.66	
F468	1.45±0.06	1.49±0.55	1.45	1.49	0.24	0.28	0.48
Y471	0.51±0.16	4.17±0.13	0.51	4.17	0.93	0.01	0.06
F487	2.65±0.11	0.74±0.30	2.61	0.57	0.00	0.00	1.00
**Y428A**							
F426	2.16±0.04	0.87±0.37	2.16	0.87	0.08	0.12	0.80
Y428							
H429	0.46±0.08	4.95±0.09	0.50	4.95	0.99	0.01	0.00
F434	0.53±0.03	3.54±0.72	0.53	3.54	0.77	0.16	0.07
F443	0.18±0.06	4.23±0.03	0.38	4.23	0.95	0.05	0.00
F446	0.56±0.04	3.87±0.06	0.56	3.87	0.85	0.07	0.08
F455	n.d.	1.79±0.74		1.79	0.32	0.68	
F466	no peak	1.32±0.31		1.32	0.20	0.80	
F468	0.43±0.16	3.20±0.18	0.43	3.20	0.68	0.30	0.02
Y471	0.21±0.10	3.24±0.07	0.38	3.24	0.69	0.31	0.00
F487	2.31±0.05	0.55±0.16	2.31	0.55	0.00	0.14	0.86
**H429A**							
F426	0.81±0.05	0.94±0.14	0.81	0.94	0.10	0.71	0.19
Y428	0.36±0.11	2.78±0.23	0.38	2.78	0.57	0.43	0.00
H429							
F434	0.47±0.11	3.41±0.16	0.47	3.41	0.74	0.22	0.04
F443	n.d.	4.13±0.03		4.13	0.92	0.08	
F446	0.52±0.09	3.87±0.05	0.52	3.87	0.86	0.08	0.06
F455	0.69±0.12	3.71±0.27	0.69	3.71	0.81	0.05	0.14
F466	no peak	1.28±0.24		1.28	0.19	0.81	
F468	1.12±0.03	0.90±0.18	1.12	0.90	0.09	0.58	0.33
Y471	0.36±0.02	3.79±0.14	0.38	3.79	0.84	0.16	0.00
F487	2.24±0.16	1.01±0.24	2.24	1.01	0.12	0.05	0.83
**F455A**							
F426	1.35±0.04	n.d.	1.35		0.57		0.43
Y428	0.44±0.07	2.44±0.27	0.44	2.44	0.49	0.49	0.03
H429	0.44±0.05	5.06±0.17	0.50	4.97	1.00	0.00	0.00

F434	0.38±0.05	3.51±0.40	0.38	3.51	0.76	0.23	0.00
F443	n.d.	4.16±0.17		4.16	0.93	0.07	
F446	0.59±0.04	3.87±0.09	0.59	3.87	0.85	0.05	0.09
F455							
F466	0.49±0.14	1.56±0.74	0.49	1.56	0.26	0.69	0.05
F468	0.67±0.05	3.03±0.28	0.67	3.03	0.64	0.23	0.13
Y471	0.21±0.07	3.73±0.12	0.38	3.73	0.82	0.18	0.00
F487	1.97±0.21	0.79±0.17	1.97	0.79	0.06	0.23	0.71
**RRM3 *wild type*/5′-UGUGU-3′**							
F426	2.49±0.06	1.93±0.66	2.48	0.80	0.06	0.00	0.94
Y428	1.13±0.25	no peak	1.13		0.66		0.34
H429	no peak	5.13±0.10		4.97	1.00	0.00	
F434	0.28±0.13	3.55±0.15	0.38	3.55	0.77	0.23	0.00
F443	0.31±0.13	4.12±0.05	0.38	4.12	0.92	0.08	0.00
F446	0.53±0.03	3.83±0.24	0.53	3.83	0.85	0.08	0.07
F455	2.65±0.32	2.47±1.18	2.45	0.85	0.08	0.00	0.92
F466	0.38±0.21	1.69±0.50	0.38	1.69	0.29	0.70	0.00
F468	0.19±0.04	no peak	0.38		1.00		0.00
Y471	0.35±0.10	3.49±0.11	0.38	3.49	0.76	0.24	0.00
F487	2.53±0.06	1.02±0.32	2.51	0.74	0.05	0.00	0.95
**RRM3 *wild type*/5′-UUUAA-3′**							
F426	1.00±0.18	n.d.	1.00		0.72		0.28
Y428	0.52±0.19	2.45±0.87	0.52	2.45	0.49	0.45	0.06
H429	0.79±0.33	5.03±0.09	0.50	4.97	1.00	0.00	0.00
F434	0.56±0.11	3.56±0.25	0.56	3.56	0.78	0.15	0.08
F443	0.43±0.08	4.05±0.07	0.43	4.05	0.90	0.08	0.02
F446	0.53±0.14	3.92±0.06	0.53	3.92	0.87	0.06	0.07
F455	0.92±0.10	3.16±0.15	0.92	3.16	0.67	0.09	0.24
F466	n.d.	2.44±0.61		2.44	0.49	0.51	
F468	0.92±0.10	1.29±0.24	0.92	1.29	0.19	0.57	0.24
Y471	n.d.	3.68±0.12		3.68	0.81	0.19	
F487	2.23±0.04	0.60±0.10	2.23	0.60	0.01	0.16	0.82
**Y428A/5′-UUUAA-3′**							
F426	2.46±0.02	1.70±0.35	2.46	0.84	0.07	0.00	0.93
Y428							
H429	0.59±0.10	5.35±0.19	0.50	4.97	1.00	0.00	0.00
F434	0.37±0.11	3.61±0.27	0.38	3.61	0.79	0.21	0.00
F443	0.19±0.15	3.97±0.10	0.38	3.97	0.88	0.12	0.00
F446	0.58±0.06	4.00±0.11	0.58	4.00	0.89	0.02	0.09
F455	1.35±0.14	1.10±0.38	1.35	1.10	0.14	0.42	0.43
F466	no peak	2.13±0.56		2.13	0.41	0.59	
F468	n.d.	3.77±0.3		3.77	0.83	0.17	
Y471	0.31±0.10	2.81±0.16	0.38	2.81	0.58	0.42	0.00
F487	n.d.	0.97±0.48		2.65	0.11	0.89	

**H429A/5′-UUUAA-3′**							
F426	1.87±0.10	1.07±0.47	1.87	1.07	0.13	0.20	0.67
Y428	1.12±0.05	no peak	1.12		0.67		0.33
H429							
F434	0.34±0.11	3.51±0.18	0.38	3.51	0.76	0.24	0.00
F443	0.24±0.06	3.87±0.11	0.38	3.87	0.86	0.14	0.00
F446	0.58±0.09	3.95±0.07	0.58	3.95	0.88	0.03	0.09
F455	1.31±0.18	n.d.	1.31		0.58		0.42
F466	2.70±0.23	1.06±0.42	2.52	0.72	0.04	0.00	0.96
F468	0.54±0.38	2.98±0.36	0.54	2.98	0.63	0.30	0.07
Y471	0.30±0.08	3.59±0.24	0.38	3.59	0.78	0.22	0.00
F487	2.45±0.01	1.27±0.21	2.45	0.85	0.08	0.00	0.92
**F455A/5′-UUUAA-3′**							
F426	2.09±0.04	1.32±0.46	2.09	1.32	0.20	0.04	0.77
Y428	0.60±0.19	2.27±0.53	0.60	2.27	0.44	0.46	0.10
H429	0.50±0.09	4.71±0.19	0.50	4.71	0.93	0.07	0.00
F434	0.36±0.10	3.44±0.20	0.38	3.44	0.75	0.25	0.00
F443	n.d.	4.13±0.09		4.13	0.92	0.08	
F446	0.51±0.09	3.85±0.13	0.51	3.85	0.85	0.09	0.06
F455							
F466	0.46±0.13	2.09±1.01	0.46	2.09	0.40	0.57	0.04
F468	0.44±0.12	3.20±0.14	0.44	3.20	0.68	0.29	0.03
Y471	0.37±0.10	2.53±0.21	0.38	2.53	0.51	0.49	0.00
F487	1.97±0.01	0.91±0.36	1.97	0.91	0.09	0.20	0.71

n.d.: not determined due to exchange-broadened peak or overlap.
no peak : not peak observable in the spin echo spectra.

Supplementary Table 3: χ1 populations across MD simulations

	χ 1	Phe426	Tyr428	His429	Phe455	Phe466	Phe468
**free CUG-BP1 RRM3 domain**							
**2rq4_1**	gauche(+)	99.1	97.5	95.3	100.0	0.0	0.0
	trans	0.1	2.5	4.7	0.0	95.9	100.0
	gauche(-)	0.8	0.0	0.0	0.0	4.1	0.0
**2rq4_2**	gauche(+)	60.9	59.2	97.7	76.5	0.0	12.1
	trans	39.1	40.8	2.3	23.5	57.8	65.9
	gauche(-)	0.0	0.0	0.0	0.0	42.2	22.0
**CUG-BP1 RRM3/(UG)$_3$ complex**							
**2rqc_1**	gauche(+)	0.0	0.0	68.5	14.4	0.0	99.9
	trans	100.0	100.0	31.5	85.7	0.1	0.0
	gauche(-)	0.0	0.0	0.0	0.0	99.9	0.0
**2rqc_2**	gauche(+)	0.0	0.0	84.5	2.1	0.0	100.0
	trans	100.0	100.0	15.5	97.9	0.0	0.0
	gauche(-)	0.0	0.0	0.0	0.0	100.0	0.0
**free CUG-BP1 RRM3 mutants**							
**2rq4_F455A**	gauche(+)	99.8	100	89.0	-	0.0	0.0
	trans	0.2	0.0	10.9	-	82.4	100.0
	gauche(-)	0.0	0.0	0.0	-	17.6	0.0
**2rq4_Y428A**	gauche(+)	100	-	97.9	100	0.7	0
	trans	0	-	2.1	0	94.7	100
	gauche(-)	0	-	0	0	4.6	0
**2rq4_H429A**	gauche(+)	100	100	-	100	0	0
	trans	0	0	-	0	96.3	100
	gauche(-)	0	0	-	0	3.7	0
**CUG-BP1 RRM3/(UG)$_3$ complex**							
**2rqc_F455A**	gauche(+)	0	0	79.2	-	0	100
	trans	100	100	20.8	-	0	0
	gauche(-)	0	0	0	-	100	0
**free CUG-BP1 RRM3 without C-terminus**							
**2rq4_Cend_removed**	gauche(+)	85.1	84.1	97.4	99.8	0.7	1.4
	trans	14.9	15.9	2.6	0.2	76.9	98.5
	gauche(-)	0	0	0	0	22.4	0.1
**CUG-BP1 RRM3/(UG)$_3$ complex without RNA**							
**2rqc_RNA_removed**	gauche(+)	89.9	90.8	99.0	99.95	0	0
	trans	10.1	9.2	1.0	0.04	85.1	99.3
	gauche(-)	0	0	0	0	14.9	0.6
**CUG-BP2 RRM3/UUUAA complex**							
**UUUAA_1**	gauche(+)	100	19.8	96.7	100	0	0
	trans	0	0	3.3	0	97.1	100
	gauche(-)	0	80.2	0	0	2.9	0
**UUUAA_2**	gauche(+)	99.9	100	98.0	100	0	0
	trans	0.1	0	1.9	0	94.2	100
	gauche(-)	0	0	0.1	0	5.8	0
**UUUAA_3**	gauche(+)	100	100	98.8	100	0	0
	trans	0	0	1.2	0	98.9	100

	gauche(-)	0	0	0	0	1.1	0
**UUUAA_4**	gauche(+)	100	100	98.9	100	0	0
	trans	0	0	0.7	0	81.3	100
	gauche(-)	0	0	0.5	0	18.7	0
**UUUAA_5**	gauche(+)	100	100	98.8	100	0	0
	trans	0	0	0.3	0	95.6	100
	gauche(-)	0	0	0.9	0	4.4	0
**UUUAA_6**	gauche(+)	100	85.7	98.6	100	0	0
	trans	0	0	1.4	0	94.8	100
	gauche(-)	0	14.3	0	0	5.2	0
**UUUAA_7**	gauche(+)	100	100	98.7	100	0	0
	trans	0	0	1.3	0	98.4	100
	gauche(-)	0	0	0	0	1.6	0
**UUUAA_8**	gauche(+)	100	100	98.6	37.4	0	0
	trans	0	0	1.3	62.6	99.9	100
	gauche(-)	0	0	0	0	0.1	0

Supplementary Table 4: RRM structural survey

A) free RRM with aromatic side-chain in UP or mixed state conformation

**PROTEIN**	**RRM**	**ORIGIN**	**METHOD**	**PDB ID**
CUG-BP1 RRM3	RRM3	HS	NMR	2CPZ.pdb, 2RQ4.pdb, 4LJM.pdb
EIF3B-RRM	RRM	HS	NMR	2KRB.pdb
EUKARYOTIC TRANSLATION INITIATION FACTOR 4B [1)]	RRM	HS	NMR	1WI8.pdb, 2J76.pdb
FUS [1)]	RRM	HS	NMR	2LA6.pdb, 2LCW.pdb
HNRNP A1 [1)]	RRM1	HS	Xray	1HA1.pdb, 1UP1.pdb, 2LYV.pdb, 1L3K.pdb
HNRNP L [1)]	RRM4	RN	Xray	4QPT.pdb, 3S01.pdb, 3TO8.pdb, 3TYT.pdb
HNRNPG/RBMX	RRM	HS	NMR	2MKS.pdb
KIAA0430 PROTEIN	RRM	HS	NMR	2DIU.pdb
NAB3-RRM	RRM	SC	Xray	2XNQ.pdb
PABPC1 A PH 9.0 [1)]	RRM2	HS	Xray	4F26.pdb, 4F25.pdb
PRP24	RRM2	SC	NMR	2GO9.pdb
RBM19	RRM5	MM	NMR	2CPF.pdb
RNA15	RRM	SC	Xray	2X1B.pdb
SC35	RRM	HS	NMR	2LEA.pdb, 2KN4.pdb
SEX-LETHAL [1)]	RRM1	DM	Xray	2SXL.pdb, 3SXL.pdb
SRSF7	RRM	HS	NMR	2HVZ.pdb
SUPPRESSOR OF CDC2	RRM3	HS	NMR	1X4E.pdb
TAR	RRM	HS	NMR	1WF0.pdb
U1A	RRM2	HS	NMR	2U1A.pdb
U2 SNRNP COMPONENT IST3	RRM	SC	NMR	2MKC.pdb, 4UQT.pdb
U2AF65	RRM3	HS	NMR	1O0P.pdb, 1OPI.pdb
UNNAMED PROTEIN PRODUCT	RRM	HS	NMR	2DIS.pdb

[1)] free RRM with multiple aromatic side-chain conformations

B) statistics

Free RRM with multiple aromatic side-chain conformations	6 (3%)
Free RRM with aromatic side-chain in UP or mixed state conformation	22 (10%)
Total number of RRM domains	188
Total number of single or tandem RRM structures*	212

* RRM domains containing at least one of the RNP aromatics.

Supplementary Table 5: ^{3}J couplings and χ1 rotamer populations: HnRNP A1 RRM1

	$^3J_{exp}$, NC$^{\gamma}$	$^3J_{exp}$, C′C$^{\gamma}$	$^3J_{calc}$, NC$^{\gamma}$	$^3J_{calc}$, C′C$^{\gamma}$	p_{-60}	p_{+60}	p_{180}
**HnRNP A1 RRM1**							
F17	2.18 ± 0.01	n.d.	2.18		0.19		0.81
F23	0.52 ± 0.12	4.07 ± 0.02	0.52	4.07	0.91	0.03	0.06
H33	3 ± 0	0.96 ± 0.24	2.73	1.09	0.00	0.00	1.00
F34	0.26 ± 0.04	3.96 ± 0.01	0.38	3.96	0.88	0.12	0.00
F37	0.17 ± 0.07	2.75 ± 0.03	0.38	2.75	0.57	0.43	0.00
F57	0.4 ± 0.15	n.d.	0.40		0.99		0.01
F59	0.08 ± 0.04	n.d.	0.38		1.00		0.00
F62	0.35 ± 0.03	3.33 ± 0.03	0.38	3.33	0.72	0.28	0.00
H77	0.24 ± 0.05	4.31 ± 0.02	0.50	4.31	0.83	0.17	0.00
**HnRNP A1 RRM1/5′-UUAGGUC-3′**							
F17	2.72 ± 0.01	n.d.	2.62		0.00		1.00
F23	0.43 ± 0.04	3.37 ± 0.07	0.43	3.37	0.73	0.25	0.02
H33	n.d.	0.94 ± 0.24		5.37	0.10	0.90	
F34	0.2 ± 0.03	3.98 ± 0.01	0.38	3.98	0.88	0.12	0.00
F37	0.25 ± 0.03	2.81 ± 0.03	0.38	2.81	0.58	0.42	0.00
F57	0.27 ± 0.03	n.d.	0.38		1.00		0.00
F59	0.16 ± 0.04	4.07 ± 0.04	0.38	4.07	0.91	0.09	0.00
F62	no peak	3.12 ± 0.03		3.12	0.66	0.34	
H77	0.17 ± 0.05	4.09 ± 0.02	0.50	4.09	0.77	0.23	0.00

Supplementary Table 6: List of RRMs exhibiting aromatic side-chain rearrangement after RNA binding

		Free				Bound						
**Protein**	**RRM**	**PDB ID**	**METHOD**	**N1 conf**	**N2 conf**	**RNA**	**PDB ID**	**METHOD**	**N1 res**	**N2 res**	**N1 conf**	**N2 conf**
NAB3	RRM	2XNQ	Xray	DWN	UP	5'-UUCUUAUUCUUA-3'	2XNR	Xray	C	U	DWN	DWN
CUG-BP1	RRM3	2RQ4	NMR	UP	UP	5'-UGUGUG-3'	2RQC	NMR	U	G	DWN	DWN
HNRNP A1	RRM1	1HA1	Xray	DWN	UP	5'-TAGGGTTAGGG-3'	2UP1	Xray	A	G	DWN	DWN
HNRNP A1	RRM1	1UP1	Xray	DWN	DWN							
HNRNP A1	RRM1	1L3K	Xray	UP/DWN	UP/DWN							
HNRNP A1	RRM1	2LYV	NMR	DWN	DWN							
HNRNPG	RRM	2MKS	NMR	DWN	UP	5'-AUCAAA-3'	2MB0	NMR	A	A	DWN	DWN
RNA15	RRM1	2X1B	Xray	OTHER	DWN	5'-UAUAUAUAAUAAU-3'	2KM8	NMR	A	U	UP	DWN
						5'-GUUGU-3'	2X1F	Xray	G		DWN	DWN
PABPC1; pH 9.0	RRM2	4F26	Xray	OTHER	DWN	5'-AAAAAAAAAAA-3'	1CVJ	Xray	A	A	DWN	DWN
PABPC1; pH 6.0	RRM2	4F25	Xray	OTHER	UP/DWN							
SC35	RRM	2KN4	NMR		DWN	5'-UCCAGU-3'	2LEB	NMR		C		UP
SC35		2LEA	NMR		OTHER	5'-UGGAGU-3'	2LEC	NMR		G		DWN
TAD BP	RRM2	1WF0	NMR	UP	UP	5'-GUGUGAAUGAAU-3'	4BS2	NMR	U	G	DWN	DWN

N conf: conformation of the aromatic side-chain in the N1 or N2 pocket
N res: Nucleotide accommodated in the N1 or N2 pocket.

Supplementary Table 7: Primers list

CUG-BP2 forward NdeI	CGG CAG CCA TAT GCA GAA GGA AGG TCC AGA GGG
CUG-BP2 bwd XhoI	GGT GGT GCT CGA GTC AGT AAG
Y114W forward	GCA AAC CTT GGT GAC TCG AGC ACC AC
Y114W bwd	GTG GTG CTC GAG TCA CCA AGG TTT GC
CUG-BP2 forward HindIII	GGT GGT GGA TCC ATG AAC GGA GC
CUG-BP2 bwd XhoI	GGT GGT CTC GAG TCA GTA AGG TTT GC
RRM12 bwd XhoI	GGT GGT CTC GAG TCA CCT TCT GAG
RRM3 forward BamHI	GGT GGT GGA TCC ATG GCG GCT C
F32A forward	GGG CAA ACC TCG CCA TTT ACC ACC
F32A reverse	GGT GGT AAA TGG CGA GGT TTG CCC
Y34A forward	GCA AAC CTC TTT ATT GCC CAC CTT CCA CAG GAA TTT G
Y34A reverse	CAA ATT CCT GTG GAA GGT GGG CAA TAA AGA GGT TTG C
H35A forward	CTC TTT ATT TAC GCC CTT CCA CAG G
H35A reverse	CCT GTG GAA GGG CGT AAA TAA AGA G
K59A forward	GTT ATC TCT GCT GCC GTC TTC ATT GAC
K59A reverse	GTC AAT GAA GAC GGC AGC AGA GAT AAC
F61A forward	CTC TGC TAA AGT CGC CAT TGA CAA ACA G
F61A reverse	CTG TTT GTC AAT GGC GAC TTT AGC AGA G
K101A forward	CAT GAA ACG CTT GGC GGT GCA GCT GAA G
K101A reverse	CTT CAG CTG CAC CGC CAA GCG TTT CAT G
Q103A forward	CGC TTG AAG GTG GCC CTG AAG CGT TCC
Q103A reverse	GGA ACG CTT CAG GGC CAC CTT CAA GCG
K105A forward	GAA GGT GCA GCT GGC CCG TTC CAA AAA C
K105A reverse	GTT TTT GGA ACG GGC CAG CTG CAC CTT C
COX-2 forward XhoI	5'-CTAGGCGACTCGAGGATCGCCGTGTAATTCTA-3'
COX-2 reverse NotI	5'-TGGCCGGCGGCCGCTATCATGTCTGCTCGAAG-3'

Supplementary Table 8: List of the simulations

simulation name	length [ns]
**free CUG-BP1 RRM3 domain**	
2rq4_1	1000
2rq4_2	1000
2rq4_Y428A[a]	300
2rq4_H429A[a]	300
2rq4_F455A[a]	300
2rq4_Cend_removed[b]	500
2rqc_RNA_removed[c]	500
**CUG-BP1 RRM3/$(UG)_3$ complex**	
2rqc_1	1000
2rqc_2	1000
**CUG-BP1 RRM3/UUUAA complex**	
UUUAA_1	1000
UUUAA_2	1000
UUUAA_3	500
UUUAA_4	500
UUUAA_5	500

UUUAA_6	500
UUUAA_7	500
UUUAA_8	500

[a]The residue mutation was introduced into the system by molecular modeling.
[b]The C-terminal residues of the protein (a.a. 105-118) were removed by molecular modeling. The simulation was started from the final structure of the 2rq4_2 simulation.
[c]The RNA molecule was removed by molecular modeling.

Supplementary Figure 1:

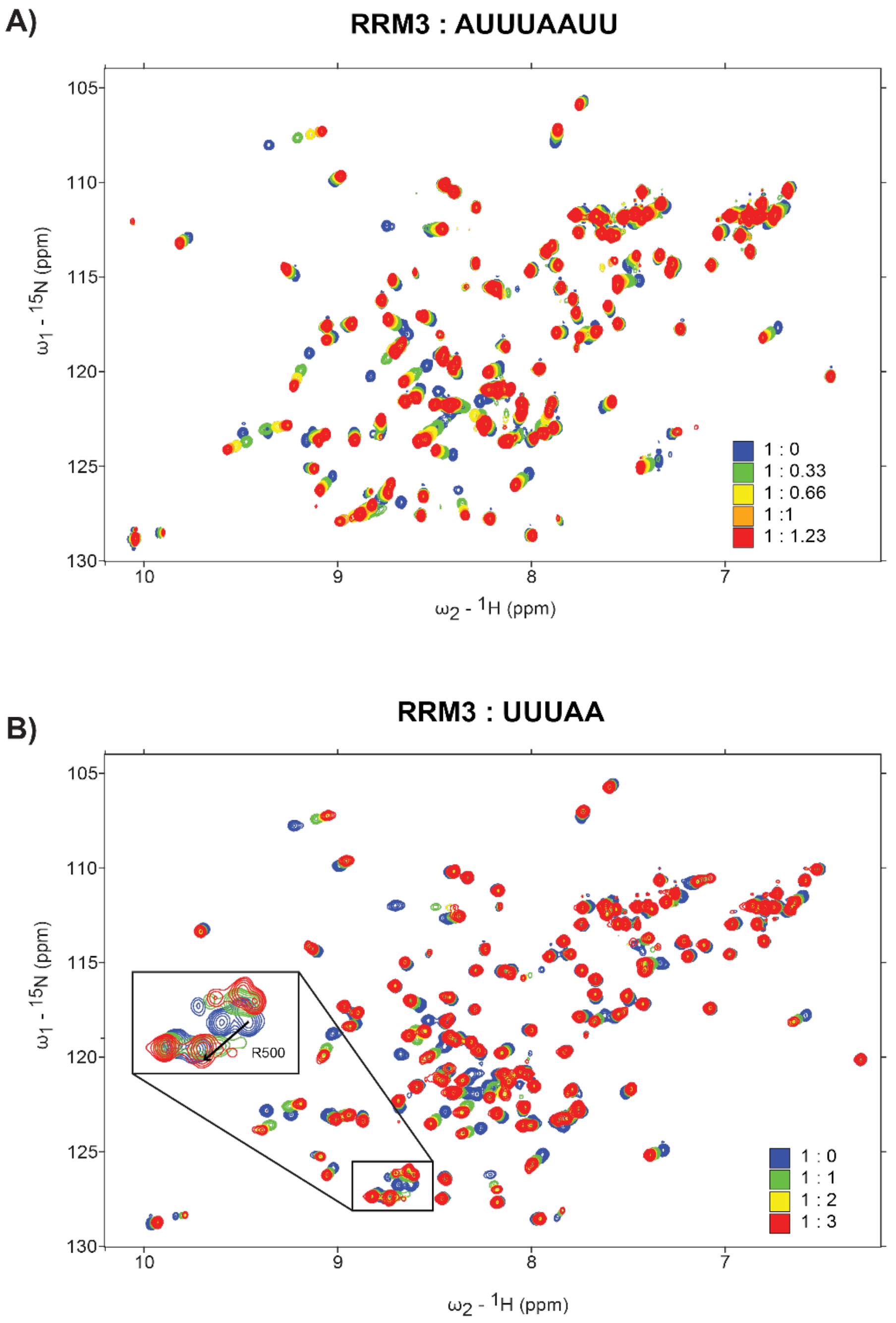

Supplementary Figure 1: ^{1}H-^{15}N 2D-HSQCs titrations of CUG-BP2 RRM3 with 5′-AUUUAAUU-3′ (upper panel) and 5′-UUUAA-3′ (lower panel). The spectra of the different titration points are overlaid and colored according to the legend in the figure.

Supplementary Figure 2:

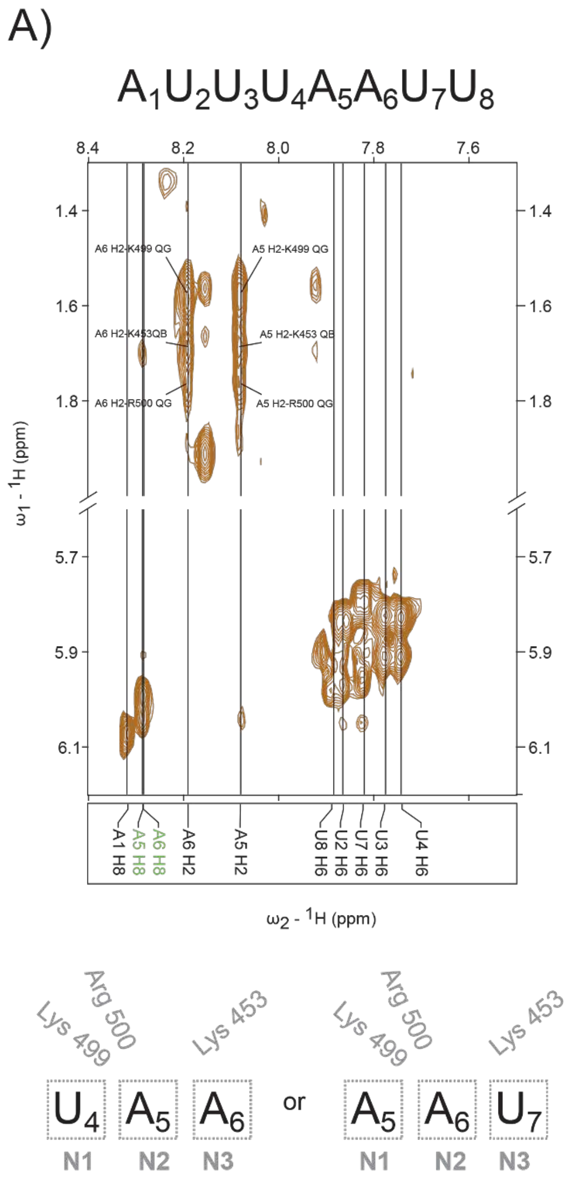

Supplementary Figure 2: AU-rich RNA binding registers. A) 2D F2 ^{13}C-filtered NOESY regions showing intra- and intermolecular NOE signals involving H6, H2 and H8 protons of 5′-AUUUAAUU-3′. The corresponding binding registers are depicted below. B) 2D F2 ^{13}C-filtered NOESY regions showing intra- and intermolecular NOE signals involving H6, H2 and H8 protons of 5′-UUUAA-3′. The corresponding binding register is depicted below. Binding pockets and protein residues are colored in gray

Supplementary Figure 3:

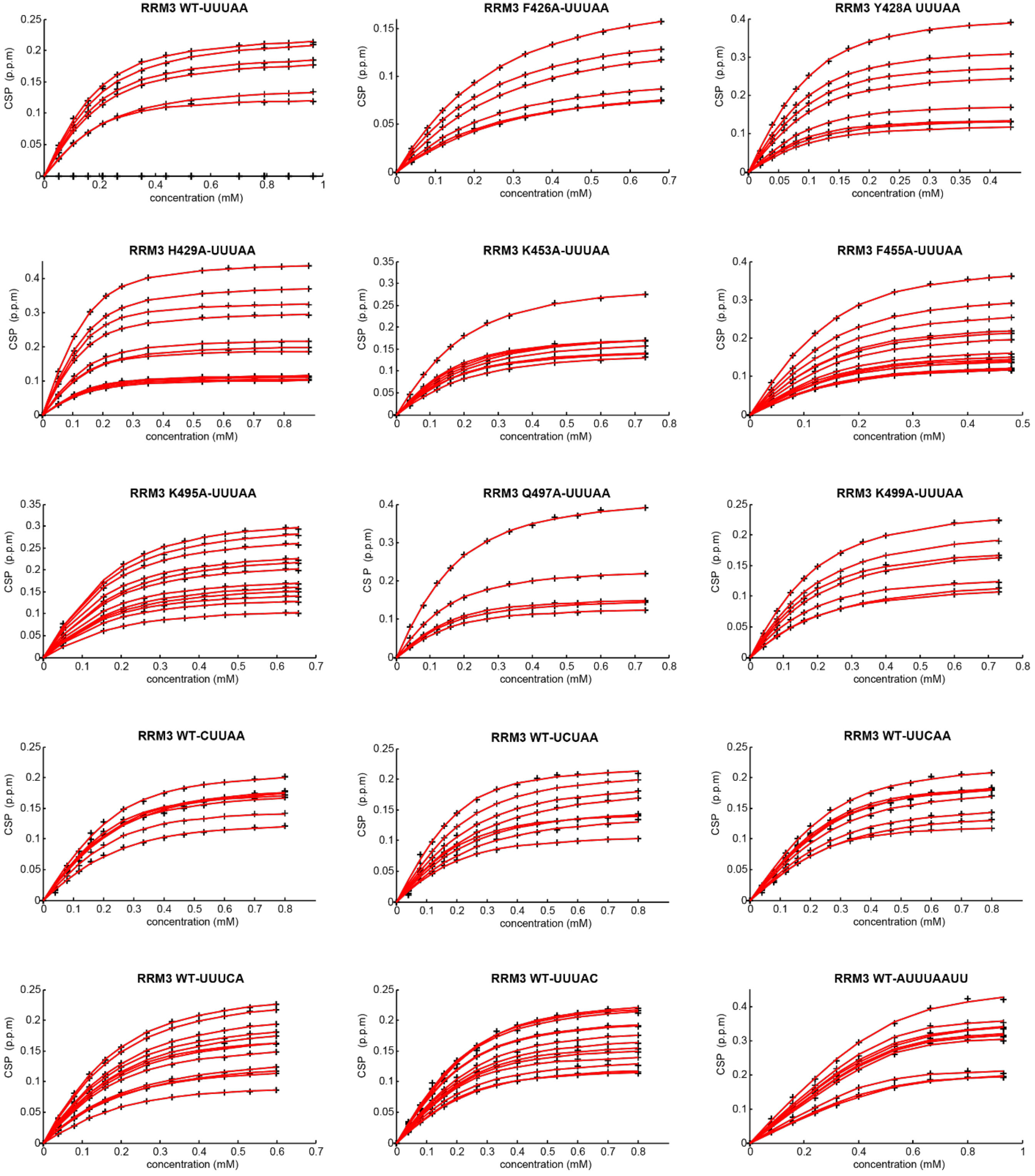

Supplementary Figure 3: ^{15}N HSQC titrations binding isotherms.

Supplementary Figure 4:

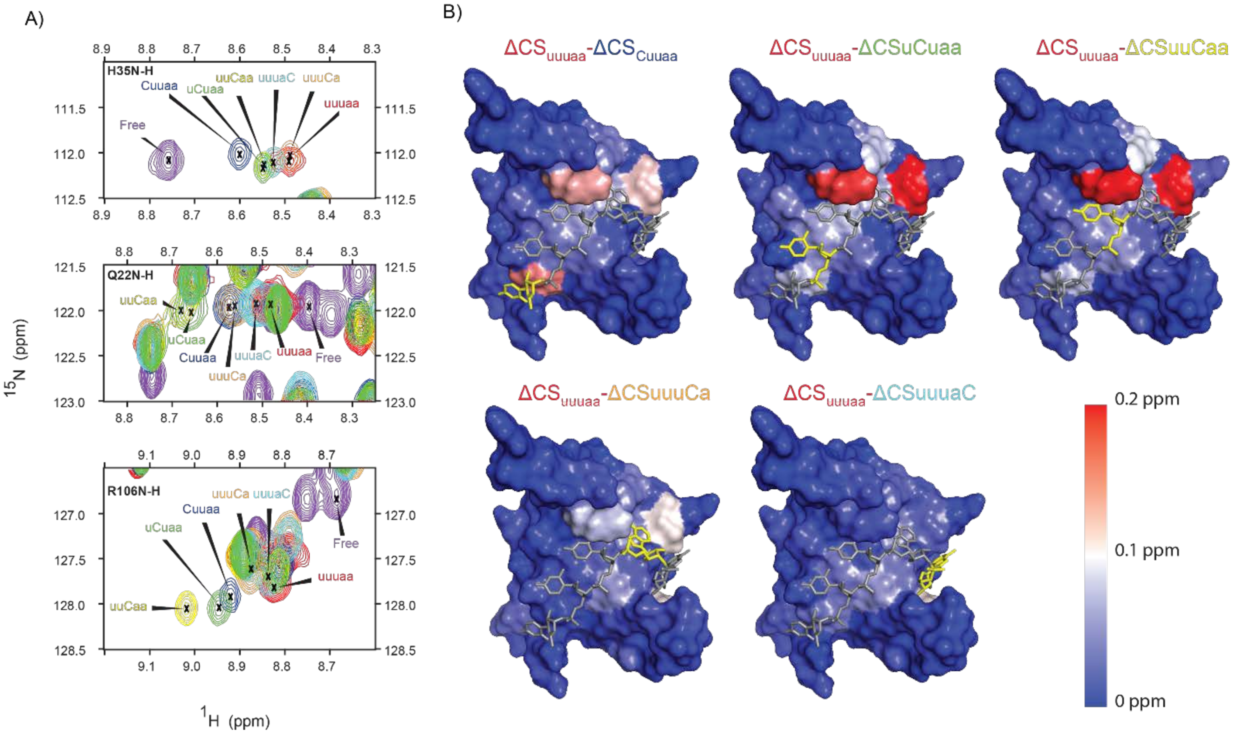

Supplementary Figure 4: Comparison of CUG-BP2 RRM3/5′-UUUAA-3′ and cytosine RNA mutant complexes. A) Overlaid portions of the ^{1}H-^{15}N 2D-HSQCs of free RRM3 (purple) and RRM3 saturated with 5′-UUUAA-3′ (red), 5′-CUUAA-3′ (blue), 5′-UCUAA-3′ (green), 5′-UUCAA-3′ (yellow), 5′-UUUCA-3′ (cyan) and 5′-UUUAC-3′ (orange). B) Mapping of the combined chemical shift difference between protein amide ^{1}H-^{15}N HSQC signals in the *wild type* and mutant RNA complexes ($\Delta CS_{wild\ type}$ - ΔCS_{mutant}) on the Van der Waals surface of RRM3 with the RNA in sticks. The mutated nucleotide is colored in yellow.

Supplementary Figure 5:

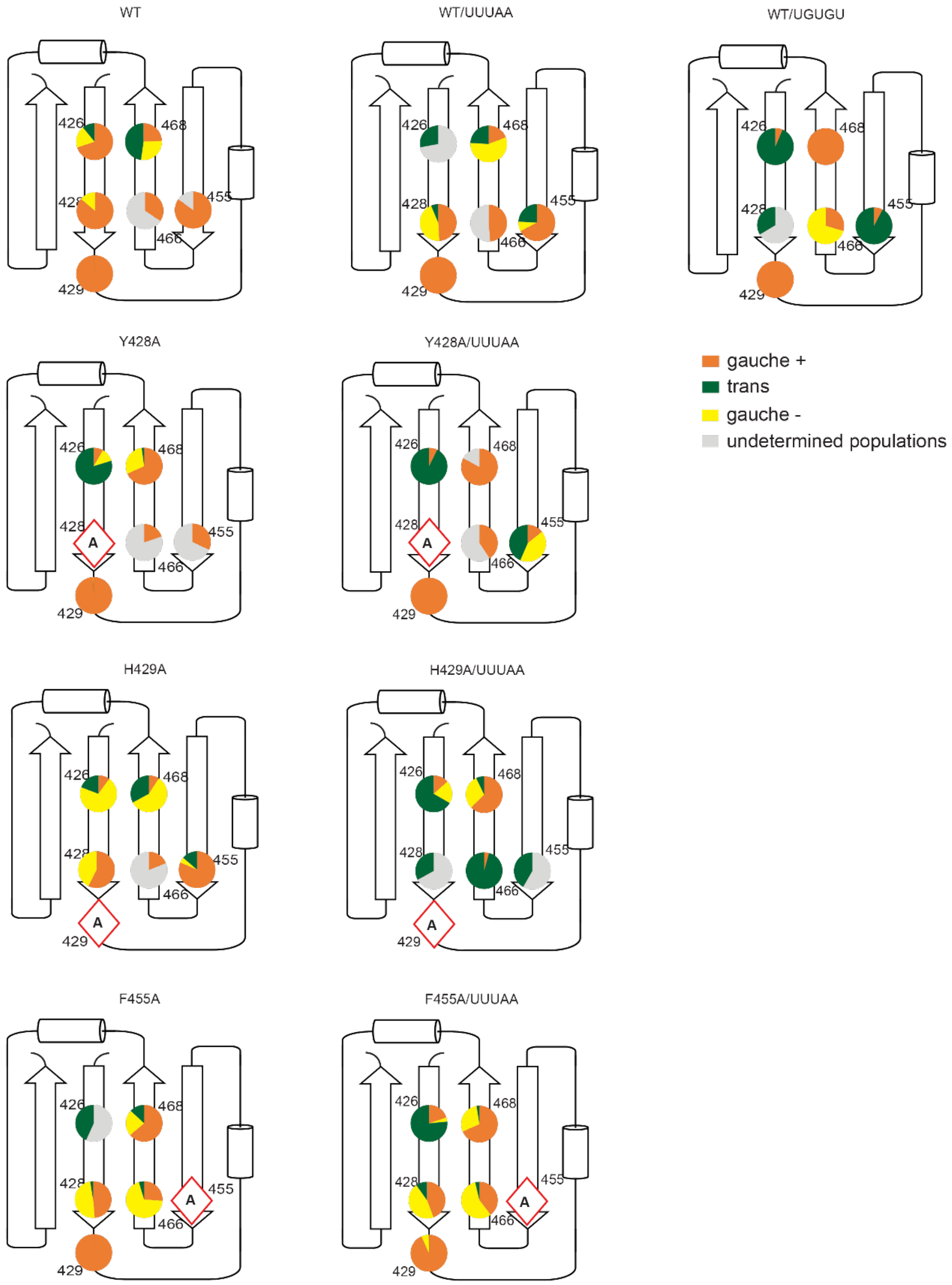

Supplementary Figure 5: Aromatic residues rotamer populations at the binding interface. RRM3 *wild type* and Y428A, H429A and F455A mutants free and bound are schematically represented with pie charts showing the $\chi 1$ rotamer populations determined from the ^{3}J NC^{γ} and ^{3}J $C'C^{\gamma}$ scalar couplings for aromatic residues at the binding interface. The populations not determined due to lack of ^{3}J coupling data are colored in grey. A red diamond indicates mutated positions.

Supplementary Figure 6

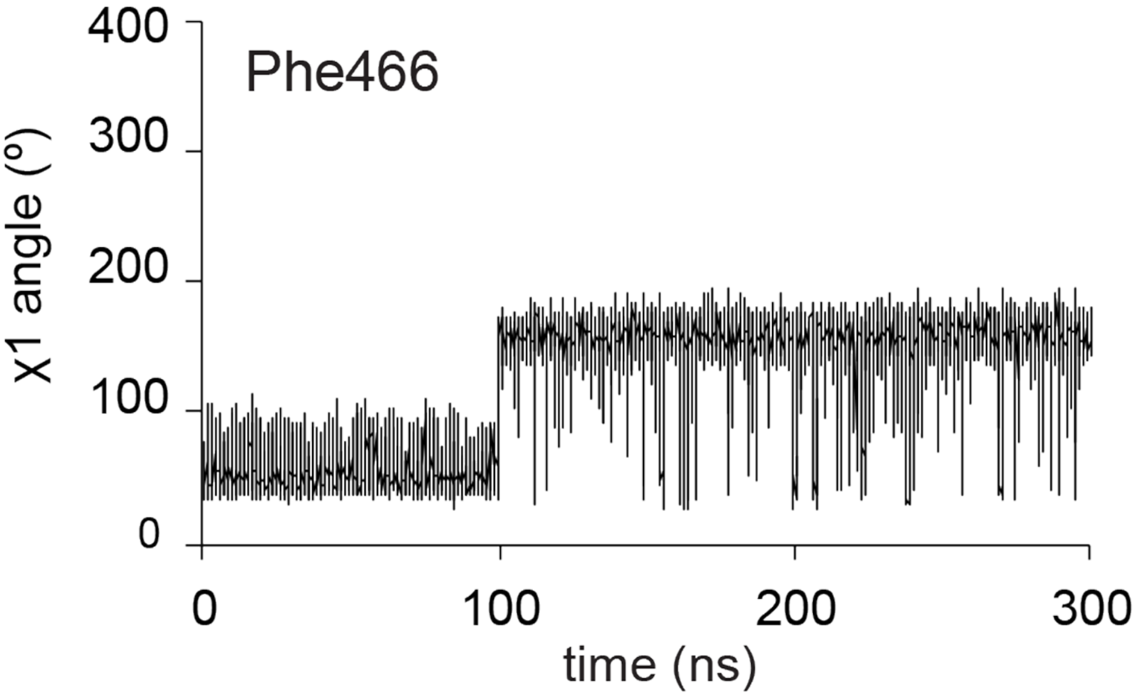

Supplementary Figure 6: RRM3 molecular dynamics simulation. Phe466 $\chi 1$ dihedral angle evolution in free RRM3 MD typical simulation.

Supplementary Figure 7:

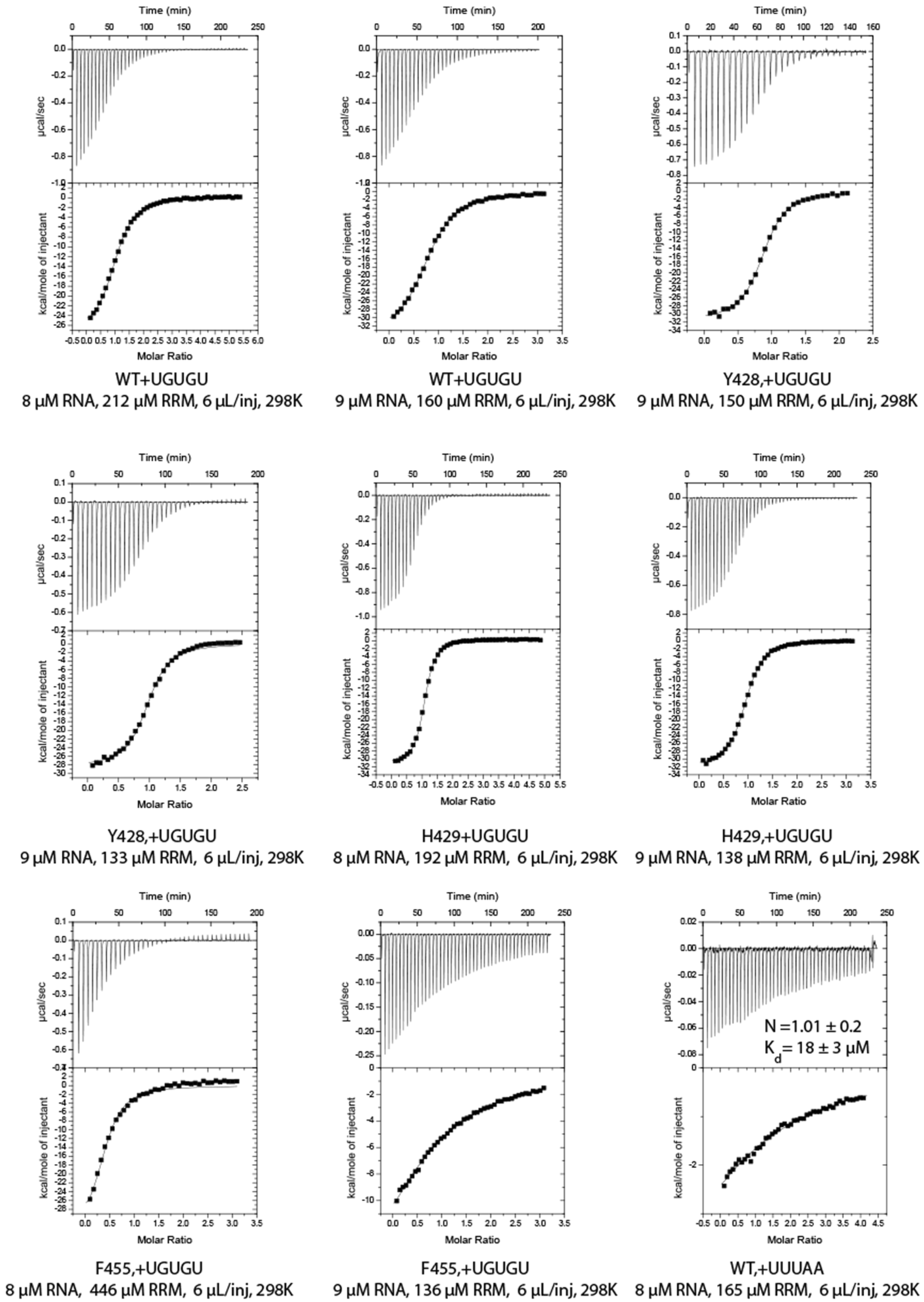

Supplementary Figure 7: ITC binding isotherms.

Supplementary Figure 8

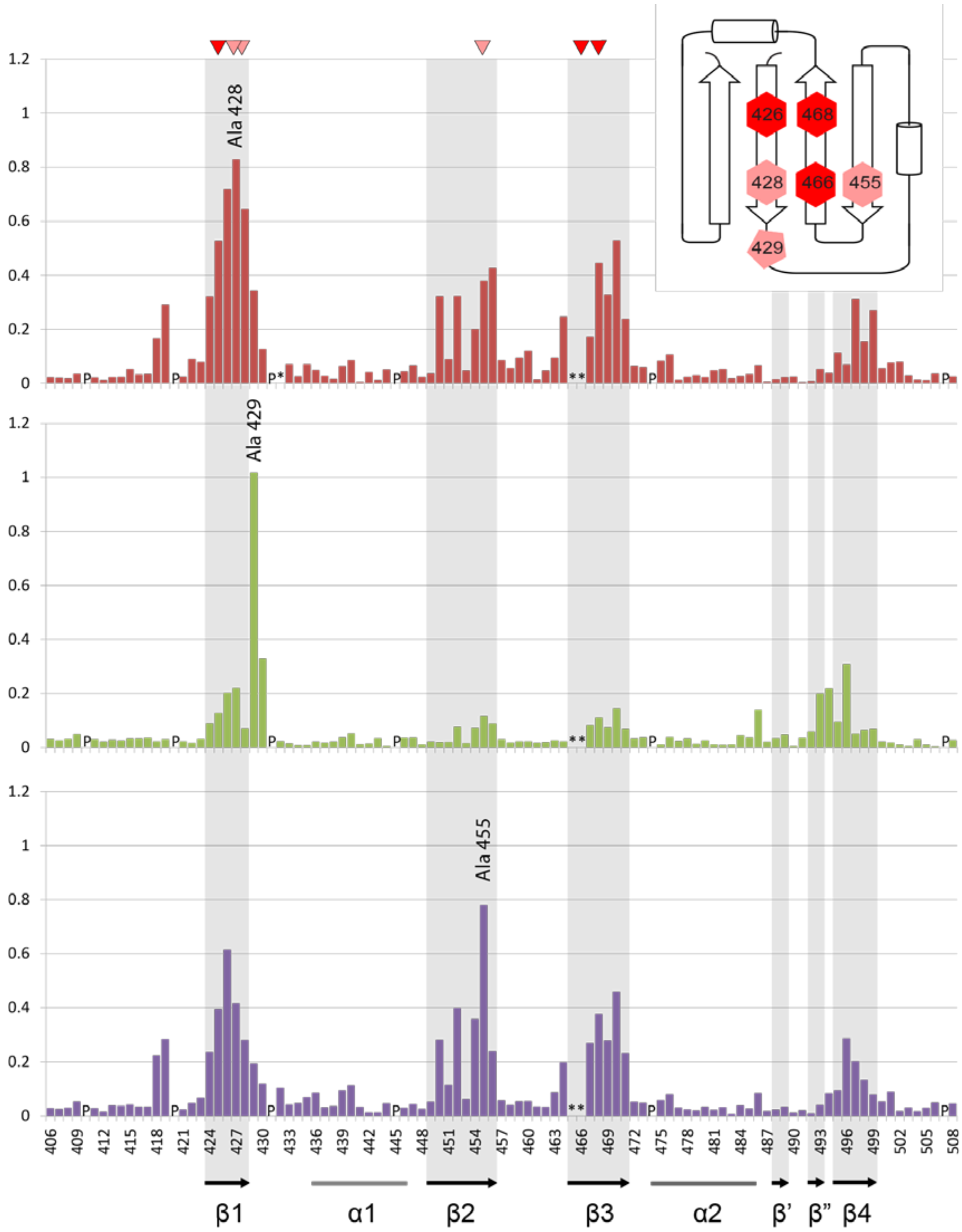

Supplementary Figure 8: Mapping of the combined chemical shift perturbations (CSP) upon mutation of an aromatic residue to alanine. The results for Y428A, H429A and F455A CSP are plotted in the upper, middle and lower panel respectively. The positions of the secondary structure elements are shown below the panel. The mutated residues are indicated. Prolines are labeled with a P and residues that could not be assigned in the mutants are designated by an asterisk. Location of aromatic residues at the binding interface are shown in a schematic diagram and by inverted triangles above the sequence.

Supplementary Figure 9:

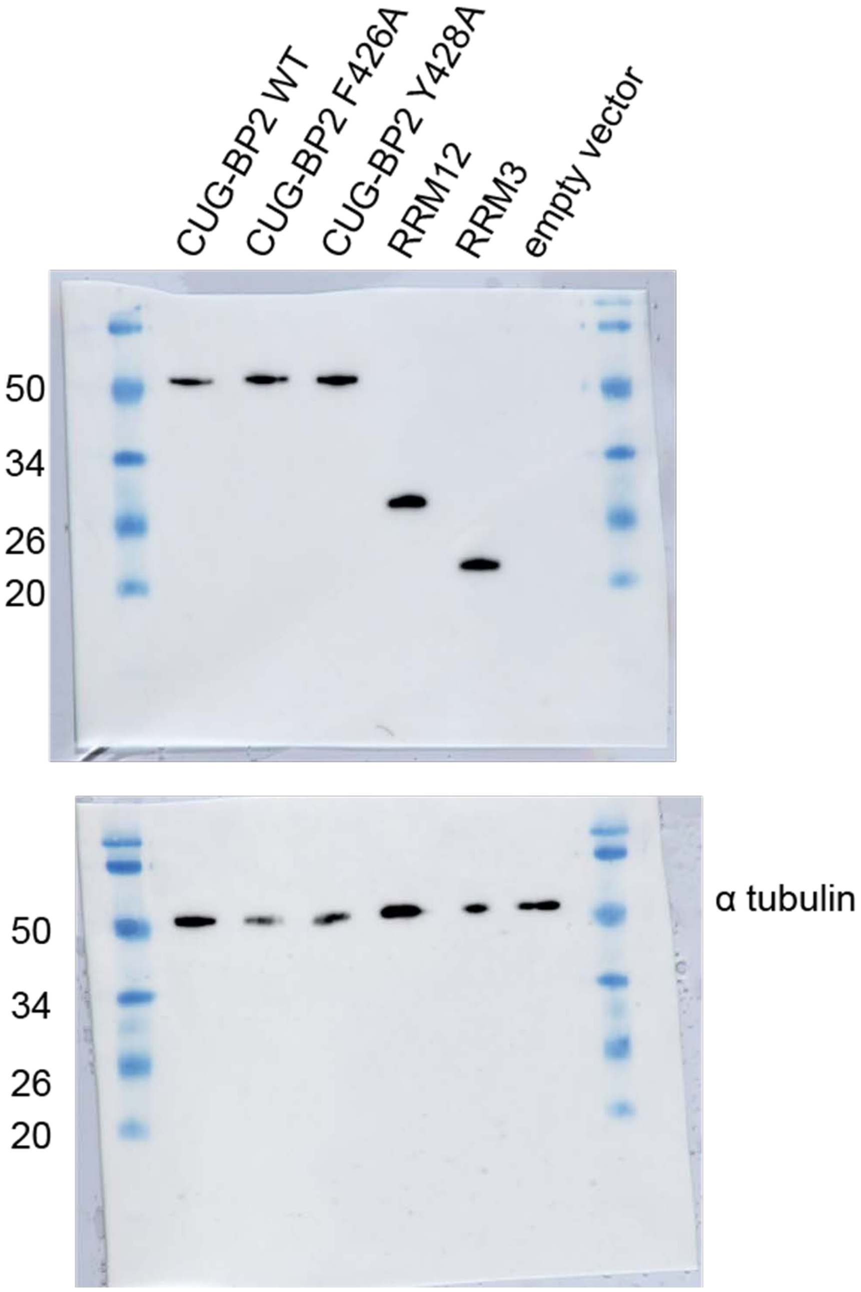

Supplementary Figure 9: Full western blots of luciferase reporter gene assay. The marker size reported on the left is in kDa.

Supplementary Figure 10:

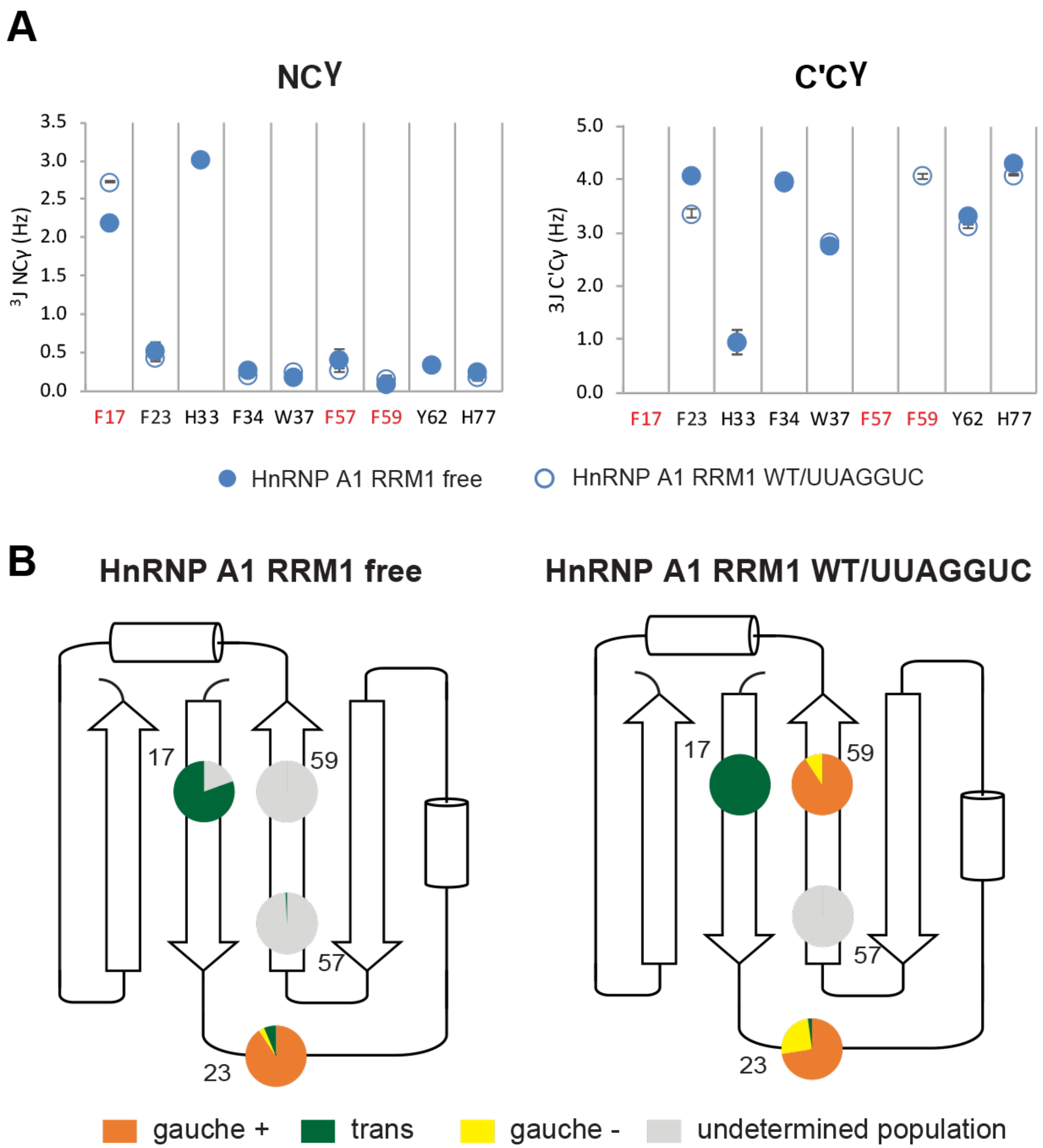

Supplementary Figure 10: hnRNP A1 RRM1 aromatic residues scalar coupling and rotamer population. A) ^{3}J NC$^\gamma$ and C′C$^\gamma$ scalar coupling values for aromatic residues of hnRNP A1 RRM1. Missing ^{3}J values are not available due to exchange broadened resonances or overlap. B) Aromatic residue rotamer populations at the binding interface. The pie chart shows the χ1 rotamer populations determined from the ^{3}J NC$^\gamma$ and ^{3}J C′C$^\gamma$ scalar coupling constants for aromatic residues at the binding interface. The populations not determined due to lack of ^{3}J coupling data are colored in grey.
